# Health Patterns Reveal Interdependent Needs of Dutch Homeless Service Users

**DOI:** 10.3389/fpsyt.2021.614526

**Published:** 2021-03-25

**Authors:** Coline van Everdingen, Peter Bob Peerenboom, Koos van der Velden, Philippe Delespaul

**Affiliations:** ^1^Department of Psychiatry and Neuropsychology, Maastricht University, Maastricht, Netherlands; ^2^Tangram Health Care consultancy, Doetinchem, Netherlands; ^3^Department of Primary and Community Care, Radboud University Medical Centre, Nijmegen, Netherlands; ^4^Department of Adult Psychiatry, Mondriaan Mental Health Trust, Heerlen, Netherlands

**Keywords:** homelessness, marginalization, mental and physical health, transdiagnostic approach, interRAI community mental health questionnaire, comorbidity rates, inclusion health, public mental health

## Abstract

**Background:** Homelessness is an increasing problem in Western European countries. Dutch local authorities initiated cross-sectional reviews to obtain accurate health and needs information on Homeless Service (HS) users.

**Methods:** The Homeless People Treatment and Recovery (HOP-TR) study uses a comprehensive assessment strategy to obtain health data. Using a naturalistic meta-snowball sampling in 2015–2017, 436 Dutch HS users were assessed. The lived experience of HS users was the primary data source and was enriched with professional assessments. The InterRAI Community Mental Health questionnaire and “Homelessness Supplement” provided information in different areas of life. The approach for mental health assessments was transdiagnostic. Raw interview data were recoded to assess health and needs. The positive health framework structured symptomatic, social, and personal health domains relevant to recovery.

**Results:** Most subjects were males, low educated, with a migration background. The majority were long-term or intermittently homeless. Concurrent health problems were present in two domains or more in most (95.0%) subjects. Almost all participants showed mental health problems (98.6%); for a significant share severe (72.5%). Frequent comorbid conditions were addiction (78%), chronic physical conditions (59.2%), and intellectual impairments (39.9%).

**Conclusion:** The HOP-TR study reveals significant concurrent health problems among Dutch HS users. The interdependent character of different needs requires an integrated 3-D public health approach to comprehensively serve symptomatic, social, and personal dimensions, required to facilitate recovery.

## Introduction

Homelessness is one of the most extreme forms of social marginalization in contemporary society (1–3). Lacking a place to live adversely affects people's life expectancy, health, autonomy, and quality of life (4–7). The literature on health and risk profiles of homeless populations reveals exclusion mechanisms, resulting in extreme inequities. The transgenerational impact of homelessness is documented in a Danish register-based cohort study (8). Fazel, Geddes, and Kushel published a critical overview article on homelessness's health consequences in high-income countries (5). Homelessness is a multi-problem issue, often including mental illness, substance abuse, and physical illness. Access to care is low, and regular homeless services fail to monitor crucial life domains such as health. More recent publications incite a dialog on social injustice and plead for inclusion health, to ascertain that socially excluded citizens also have access to the highest care standards (9).

For several reasons, knowledge of health problems in homeless populations is fragmented and incomplete. First, the welfare system is the primary care network for homeless people. Medical records are unavailable since professionals assess people without knowledge or skills to systematically collect and interpret health information. Second, homeless people have other survival priorities. Financial problems often interfere with care access and engagement. Third, traditional censor data often use addresses as sampling frames. Consequently, people without an address are excluded from monitoring and underrepresented in health surveys. Finally, the categorical nature of dominant psychiatric classifications (such as the Dsm-5 system) hinders the collection of relevant mental health data (10). As a result, information on comorbid conditions and other needs relevant to recovery is masked.

Scientific evaluations of homelessness interventions in the past decade increasingly comprised indicators of behavior, quality of life, resilience, and personal perceptions (11–13). Still, mental health data are mainly collected from the perspectives of healthcare professionals. The literature on health problems in homeless populations that combines both HS users' and professional views is scarce.

In the Netherlands, the number of homeless people has doubled since 2009 (14). Recent monitoring data on homelessness is unavailable after discontinuing the National Strategy Plan for Social Relief in 2014 (15, 16). The figures of Netherlands Statistics (CBS) only include the most visible, nuisance-giving roofless part of the Dutch homeless population (17). By contrast, the definition of the European Typology of Homelessness and housing exclusion (ETHOS) is broader and contains people who are roofless, houseless, or living in insecure or inadequate homes (18). Thus, CBS statistics underestimate the prevalence compared to ETHOS categories. CBS health data (19) are limited to the care prevalence of mental health issues, as registered in insurance declarations (in 2012–2016; present in 46%).

The CODA-G4 study^1^, a multisite cohort study for monitoring the Dutch national action plan to end homelessness (2011–2013), collected health data on Homeless Service (HS) users. The cohort was designed to identify predictors of quality of life and stable housing (20, 21). More than 500 homeless people participated in the initial assessments, that included health using inventories and questionnaires. Compared to the general population, the homeless people scored high on somatization, depression, and anxiety on the Brief Symptom Inventory-18 (22). In the adult HS user group (23 years and older), 42.7% reported cannabis use, and 28.5% drinking five or more alcoholic drinks at one occasion in the previous month. Substance use was absent in 42.3%. Intellectual disability was suspected in 29.5% of cases (23). The results for young and older adults were described separately. Comorbid overlap was unavailable. The only documented comorbidities were Intellectual Disability (ID) and substance use. Half the persons with suspected ID also reported regular substance use within the last 12 months (51.8%).

The HOP-TR study was initiated by local authorities to provide service planning information. The primary study objective was to optimize services and enable sustainable recovery of marginalized individuals with complex needs. An integrated assessment strategy was developed to collect relevant health data. It contains a broad assessment of health aspects in different areas of life. The screening focus shifts from marginalization trajectories to recovery processes. Human rights provided context to assess health and needs. Different domains are reviewed to gain insight into the interaction of homeless people with care networks. The positive health framework structures the data domains and their mutual relations: symptomatic (physical and mental), social, and personal health (24, 25). A complete description of the design and assessment approach was described in a separate paper (26). This paper presents the results in this study on the symptomatic health domains.

## Methods

### Design and Participants

The HOP-TR study was initiated to fulfill local needs for accurate management information on HS users' health and needs. Between March 2015 and November 2017, a multistage cross-sectional design was used to collect data on Dutch HS users in different shelters and homeless services. A double snowball sampling was used to obtain data: sampling of settings and sampling of individuals within settings. In the selection of consecutive locations (naturalistic meta-snowball of services), the regional spreading and facility types were monitored until saturation occurred. In each facility, a participatory approach was used to recruit the original HS users (snowball sampling of individuals). They were asked to name similar subjects and these were also interviewed until the sample was representative. Sixteen facilities in seven cities and 436 HS users participated in the study.

### Instruments

A comprehensive assessment strategy was developed to collect health data in semi-structured interviews. All interviews were conducted by an independent researcher with a professional background as a medical doctor (first author: CvE). The lived experience of HS users was the primary source of data. Additional professional assessments were added. Open questions were used to collect personal biographies, including homelessness history, social context, care history, and personal goals. The ETHOS Typology of all subjects was assessed (18).

The interRAI Community Mental Health questionnaire [CMH; (27, 28)] and the Homelessness Supplement were employed to define indicators for different life domains. For instance, the CMH variable “Cognitive skills for daily decision making” takes all mental health issues and related behavior into consideration; the quality and safety of daily decisions relates to the presence and degree in which supervision is needed. On indication, cognitive and intellectual disability screeners were used (26).

Collected data were entered in digital case report forms. The CMH algorithms were applied to calculate the CMH scales and Clinical Assessments Protocols (CAPs). Interview data were structured in the health domains of the positive health framework. Physical health data include symptoms, functioning, disease, and duration (chronic status). Mental health data comprise mental state indicators, traumatic life events, substance use and behavior, cognition, intellectual impairments, transdiagnostic features, and (HS user's) information on mental health diagnoses.

Interview data on physical and mental health, care use, and the CMH CAPs and scales were reviewed to assess recoded variables. The recoded variables were considered necessary to assess modern state-of-the-art health and care needs in a rights-based, recovery-oriented perspective (26). The transdiagnostic features of mental health are the main recoded variables in this paper. A transdiagnostic approach evaluates clinically significant complaints or behaviors (vulnerabilities and symptoms), which characterize the current health problems and the course of subjects' disability (10, 26, 29, 30). Only current transdiagnostic features were scored. Past symptoms and vulnerabilities which do not impact actual daily functioning were not reported. A decision tree based on the Dutch consensus definition of Serious Mental Illness EPA^2^ was added to summarize the presence and character of mental health-related needs (31). The probability estimates of alcohol abuse were based on the peak number of alcoholic drinks (five or more at one occasion in the last 2 weeks).

### Analysis

The information about different settings and sources was integrated into one database. Statistical analyses were done in Stata 13. Most consisted of descriptive analyses. For example, frequencies and percentages reflect prevalence estimates of physical disorders or the overlap in substance use patterns. Additionally, chi-square and *t*-tests were run to compare the demographic features of the sample to the CODA-G4 and official CBS homelessness figures. They assess the representativeness of our sample.

### Ethical Statement

The commissioning organizations gave permission for the scientific use of the management-data. The research ethics committee of the Radboud University Nijmegen Medical Center certified that the research does not fall within the remit of the Medical Research Involving Human Subjects Act (file number 2018-4463). Individual subjects were informed about the study aims and provided informed consent. A small reward was provided.

## Results

The interviews provided the raw data on the health results presented in this section.

Unless stated differently, all numbers are percentages. All percentages express the share of the total sample in which the feature was observed.

### Sample Profile

Table 1 portrays the background characteristics of the study sample.

**Table 1:** Background characteristics of the sample (in %).

Gender	Male	81.0
	Female	19.0
Age	18–29	19.3
	30–49	46.6
	50–64	29.6
	65 or older	4.6
Migration background	Netherlands	47.9
	Other western countries	13.1
	Non-western countries	39.0
	First generation	39.2
	Second generation	12.8
Education: highest level completed	Low	82.3
	Middle	14.9
	High	2.8
European typology on homelessness and housing exclusion (ETHOS)	Roofless: rough sleepers	7.6
	Roofless: night shelters	67.4
	Houseless: in homeless accomodation	21.1
	Houseless: long term homeless supported living	1.8
	Houseless: independent living with long term support	2.1
Homelessness history	Previous homelessness (ETHOS)	78.8
	Residential instability in past 2 years	91.7

Most subjects were males, low educated, with a migration background. Their mean age was 42.9 years (males 43.1; females 41.8; range 18–75). All HS users met the ETHOS criteria. Most HS users were roofless (75.0%): they had slept rough or in night shelters. A smaller share was houseless (25.0%): those subjects had a place to sleep in crisis shelters, hostels or supervised appartments. The high proportions on “previous homelessness” and “residential instability” indicate the long-term or intermittent character of homelessness. Only few HS users had a regular job (2.8%). Financial problems were common. Two out of three perceived obstructions in the purchase of essential goods in the last month (65.8%). Some residents had no income, and were not compensated by a social security allowance (14.9%). One out of four did not have a health insurance at the moment of the interview; four out of five (79%) had not visited any physician within the last 3 months.

The sample characteristics of the HOP-TR sample were compared with reference data [CODA-G4 and CBS 2015-2017; (22, 32)] to assess its representativeness (Supplementary Tables 1, 2). No differences were found for gender, but subjects in the HOP-TR sample were significantly older and had less frequently a migration background. Compared to the CBS samples, the education level of subjects in the HOP-TR sample was lower.

### Health

Half the subjects considered their health as sufficient: excellent (5.7%) or good (47.5%); half as insufficient: fair (27.8%) or poor (18.8%). Results regarding physical and mental health are described. Additionally, symptoms related to behavior, social interaction and functioning in daily life are presented.

#### Physical Health

The left column of Table 2 shows the presence of common physical symptoms. 51.6% of respondents report fatigue, 32.8% pain, and 26.4% gastrointestinal complaints. Emergent conditions, such as fever or injuries, were present in 12.8%.

**Table 2:** Physical health status (in %).

**Physical symptoms**	**Current presence**	**Total presence**	**Chronic physical conditions**	**Presence**
Headache	12.6	17.9	Neurological	12.6
Dizzyness	8.3	12.2	Visual	3.9
Acid reflux	7.6	10.8	Auditory	3.4
Nausea	4.1	5.3	Endocrine	6.9
Vomiting	2.8	3.5	Gastrointestinal	13.5
Constipation	5.3	6.7	Infectious	2.8
Diarrhea	2.8	4.1	Respiratory	9.2
Blurred vision	3.9	4.4	Cardiovascular	12.2
Dyspnea	16.7	16.7	Musculoskeletal	0.9
Chest pain	2.5	6.2	Malignancy	1.8
Peripheral edema	2.5	3.0	Under-/overweight	41.7
Difficulty urinating	10.6	11.7		
Skin problems	9.2	9.2		
Foot problems	13.1	13.1		

*The left column depicts the presence of common physical symptoms in the past 3 days and in total. The right column shows the prevalence estimates of chronic physical conditions*.

The right column shows prevalence estimates of physical disorders. The results depict severe chronic physical conditions, related to higher morbidity, mortality, and impairments rates. Chronic conditions of the gastro-intestinal (13.5%), neurological (12.6%) and cardiovascular (12.2%) dirorders are most prevalent. Gastrointestinal disorders include esophagitis or gastritis (7.3%), chronic liver disease (5.7%), and chronic pancreatitis or m. Crohn (0.5%). Neurological disorders comprise polyneuropathy (8.3%), stroke (2.3%), and a rest group including severe conditions such as multiple sclerosis and epilepsy (2.1%). Cardiovascular disorders consist of hypertension/hypercholesterolemia (7.1%), chronic heart disease (4.6%), and intermittent claudication (0.5%).

Chronic conditions of the respiratory, endocrine and infectious disorders are less prevalent. Respiratory disorders taper to asthma and COPD (9.2%). Endocrine disorders (6.9%) involve diabetes (5.5%) and thyroid disease (1.8%). Infectious disorders include viral hepatitis or HIV (2.8%).

Impairments in the ability to see (3.9%) and to hear (3.4%) are rarely observed. The prevalence of chronic musculoskeletal disorders is low (0.9%), since it is tapered to leg amputations and to chronic musculoskeletal conditions causing severe impairments. Malignancies are scarcely reported (1.8%). Instead, weight abnormalities were most prevalent (41.7%), and usually are related to overweight (36.2%), rarely underweight (5.5%).

#### Mental Health

**Figure 1:**
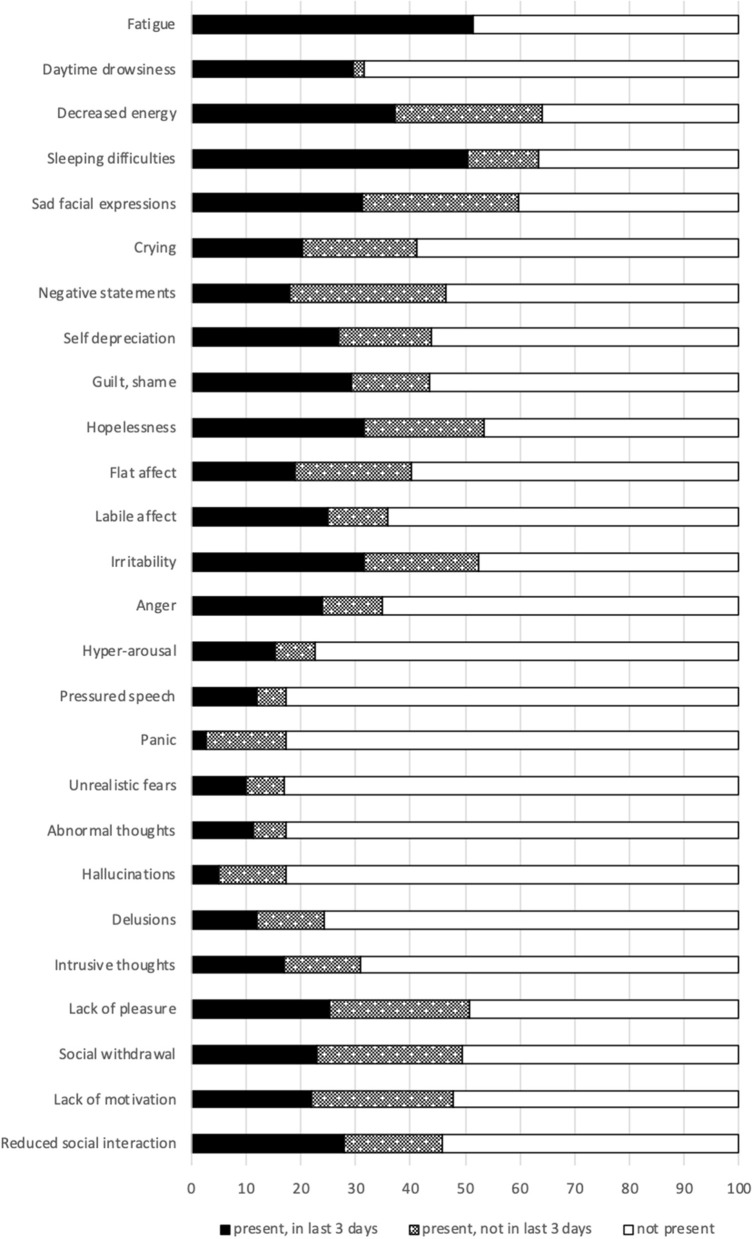
Mental health status. SR indicates Self-Reported mood symptoms.

Figure 1 depicts symptom frequencies on mental health. The Self-Reported (SR) mood reflects the character and severity of perceived mental health symptoms. Most subjects experience anxiety (66.1%), sadness (54.4%), or loss of interest (51.1%). Also, subjects report decreased energy (64.0%), hopelessness (53.4) or irritability (52.3%). Intrusive thoughts are observed in one out of three (31.0%). Panic and unrealistic fears are less common (both 17.2%).

Psychotic symptoms were prominent. Hyperarousal (22.7%), delusions (24.3%), abnormal thoughts (17.2%), and hallucinations (17.4%) are most common. Command hallucinations were reported less frequently (4.8%). The Positive Symptoms Scale scale assesses the presence and severity of psychotic symptoms. One out of two (46.3%; mean score 3.8; range 0–12) had a positive score.

Patterns in mental health features were assessed in a transdiagnostic approach. Transdiagnostic features reflect the presence of major complaints and behaviors, which qualify current mental health. Therefore, the transdiagnostic features in Table 3 are prevalences over the past 3 months. Addiction is the most prevalent (78.0%): prior use (15.1%) and active substance use (62.8%).

**Table 3:** Prevalence estimates of transdiagnostic features (in %).

Addiction	78.0
Anxiety	75.2
Trauma	69.3
Depression	67.0
Psychosis	30.5
Agitation or aggression	64.2
Problematic personality	64.5
Intellectual impairments	39.9
Neurocognitive impairments	28.4
Somatization	17.4

Similarly, the transdiagnostic features anxiety (75.2%), trauma (69.3%), and depression (67.0%) are frequently present. At times, a psychotic vulnerability is observed (30.5%). Agitation (64.2%) and personality problems are common too (64.5%). More than one third of respondents had intellectual disabilities (39.9%) or neurocognitive impairments (28.4%). Occasionally, transdiagnostic features of somatization are present (16.7%).

A decision tree based on the Dutch consensus EPA was used to assess the presence and character of care needs related to mental health problems. In one out of twenty (5.3%) mental health related care needs were absent. Subjects had no mental problems or displayed a sufficient personal resilience and social support to overcome the mental problems observed. A significant part needs mental care, but no longterm multidisciplinary care (22.3%) – a requirement in the Dutch EPA criteria. Most (72.5%) subjects in the HOP-TR study do have a long-term need of integrated multidisciplinary care, because of the pervasive nature and circular character of the mental illness and social disabilities.

#### Comorbidity

Cumulative numbers and overlap of concurrent health problems over different domains are presented in Table 4. Panel 4A presents the number of transdiagnostic mental health features, physical conditions, and the number of health domains affected. Almost all subjects have some health problems (99.3%). Transdiagnostic mental health features are highly prevalent (98.6%), while physical health problems are common (59.9%).

**Table 4:** Cumulative numbers and overlap of concurrent health problems (in %).

**Cumulative numbers**		**Mental health: transdiagnostic features**		**Physical conditions**		**Health domains**	
		**(range: 0–10)**		**(range: 0–6)**		**(range: 0–4)**	
		**Absent**	**Present**	**Absent**	**Present**	**Absent**	**Present**
Any		1.4	98.6	40.8	59.9	0.7	99.3
One			2.1		31.7		4.4
Two			3.9		14.4		33.0
Three			8.9		9.2		42.4
Four or more			83.7		4.6		19.5
**Overlap**		**Mental illness**		**Addiction**		**Intellectual impairments**	
		**Absent**	**Present**	**Absent**	**Present**	**Absent**	**Present**
Addiction	Absent	2.1	20.0				
	Present	0.0	**78.0**				
Intellectal impairments	Absent	1.4	58.7	14.7	45.4		
	Present	0.7	**39.2**	7.3	**32.6**		
Physical conditions	Absent	0.9	39.2	7.3	32.8	23.9	16.3
	Present	1.1	**58.7**	14.7	**45.2**	36.2	**23.6**

*Addiction and intellectual impairments are included in the transdiagnostic features in panel 4A; they are not included in ‘mental illness' in panel 4B. The right column of panel 4A shows the number of health domains affected (mental illness, addiction, intellectual impairments and physical disease). Panel 4B presents the overlap of the various health domains in bold*.

Nearly all subjects had health problems in two or more health domains (95.0%). In six out of ten, health problems are present in three or four domains (61.9%). The combination of mental illness with addiction is most frequent (78.0%). Chronic physical conditions nearly always occurred as a comorbid condition with mental illness or intellectual impairments.

#### Behavior and Daily Performance

**Figure 2:**
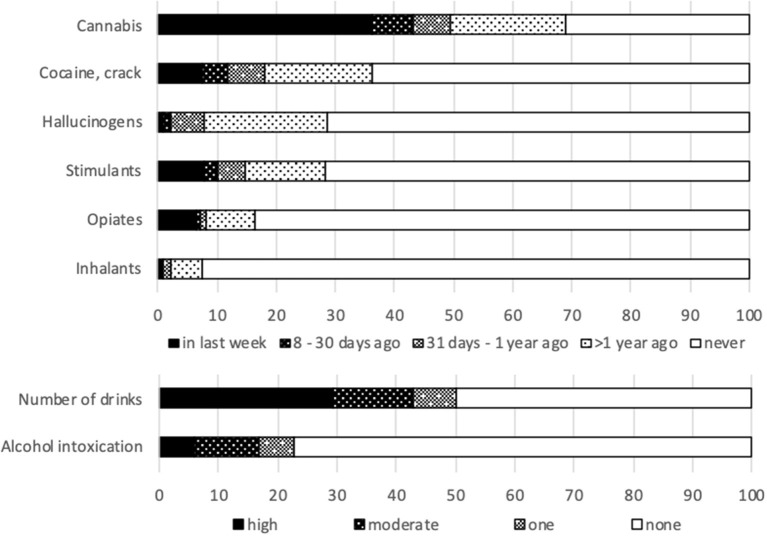
Substance use frequency. Number of alcoholic drinks in the last fortnight: one; 2–4 (moderate); 5 or more (high). Number of days to point of intoxication in the last month: one; 2–8 (moderate); 9 times or more (high).

Table 5 and Figure 2 present the results on substance use. Over the previous month, 60.6% reported any substance abuse. Alcohol abuse (five drinks or more at one occasion) was reported by 29.4%. Recurrent drunkenness was 10.8% for 2–8x/month and 6% for 9 or more. 50.5% reported drug use over the past month.

**Table 5:** Substance use patterns (in %).

	**Never**	**Ever**	**Last year**	**Last month**	**Last week**
Daily tobacco use					74.5
Cannabis use	31.2	68.8	49.5	43.1	36.2
Hard drugs use	55.7	44.3	28.2	20.6	16.3
Injecting drugs use		4.6		0.2	
Drugs use	28.7	71.3	56.0	50.5	43.1
Alcohol abuse				29.4	
Any substance use				60.6	
No substance used				39.5	

*Frequencies of substance use, expressed in percentages as proportions of the whole sample. No alcohol abuse is defined as having used no alcohol (5 drinks or more at one occasion in the last 2 weeks). No substance used is defined as having no alcohol abuse and no cannabis or hard drugs in the last month*.

**Figure 3:**
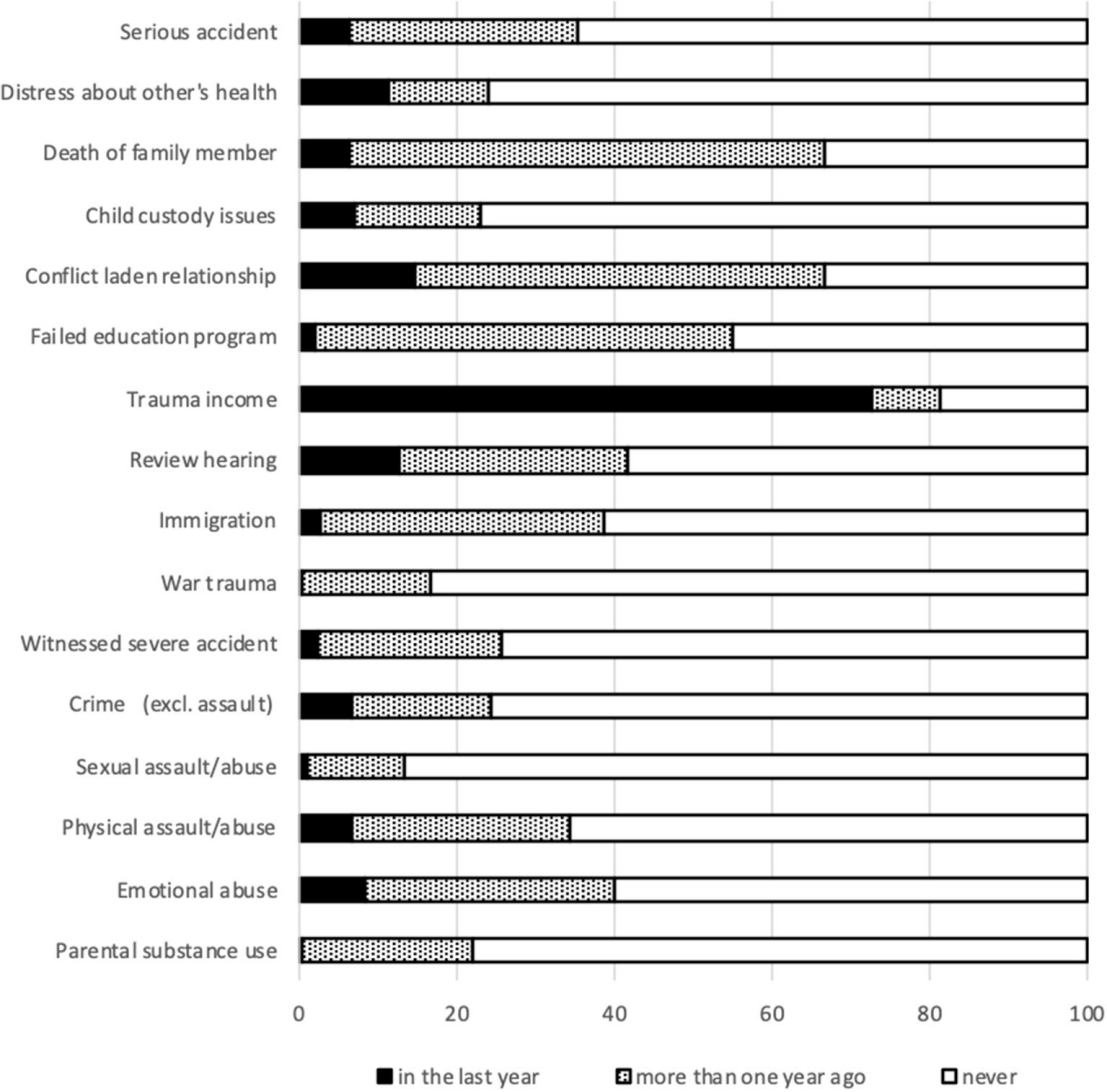
Lifetime prevalence of trauma.

Figure 3 portrays the lifetime prevalence of trauma. Almost all subjects (99.1%) reported one or more lifetime traumas (mean 6; range 0–14). Income related traumas are most common: financial troubles and income uncertainty occurred in 72.7% over the last year.

**Figure 4:**
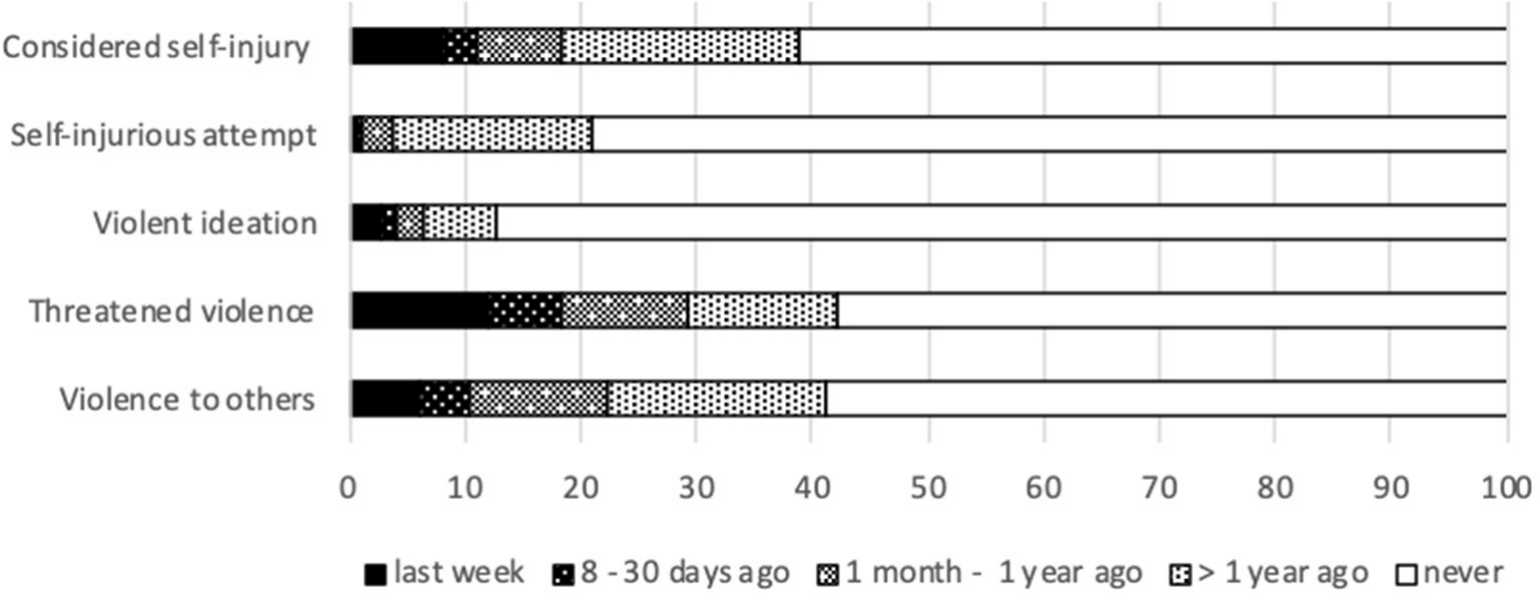
Indicators of violent behavior.

Prior traumas still have significant impact on daily life. A substantial part of subjects reported intrusive thoughts (31.0%), intense fear (34.9%) or immediate safety concerns (7.3%). Figure 4 presents the results on indicators of violent behavior. One out of ten subjects considered self-injury in the last month (11.4%) and 10.3% exhibited violent behavior.

With respect to cognitive skills, concentration loss was observed in 22.7% of the subjects. 35.1% had short-term memory problems and 7.3% procedural memory problems. Most subjects were capable of making independent, safe, and reasonable decisions (58.9%). Some have problems in new situations (8.0%). By contrast, a significant number (22.0%) recurrently made poor or unsafe decisions, and one out of 10 needed structural supervision due to impaired daily decision making (10.8%).

**Figure 5:**
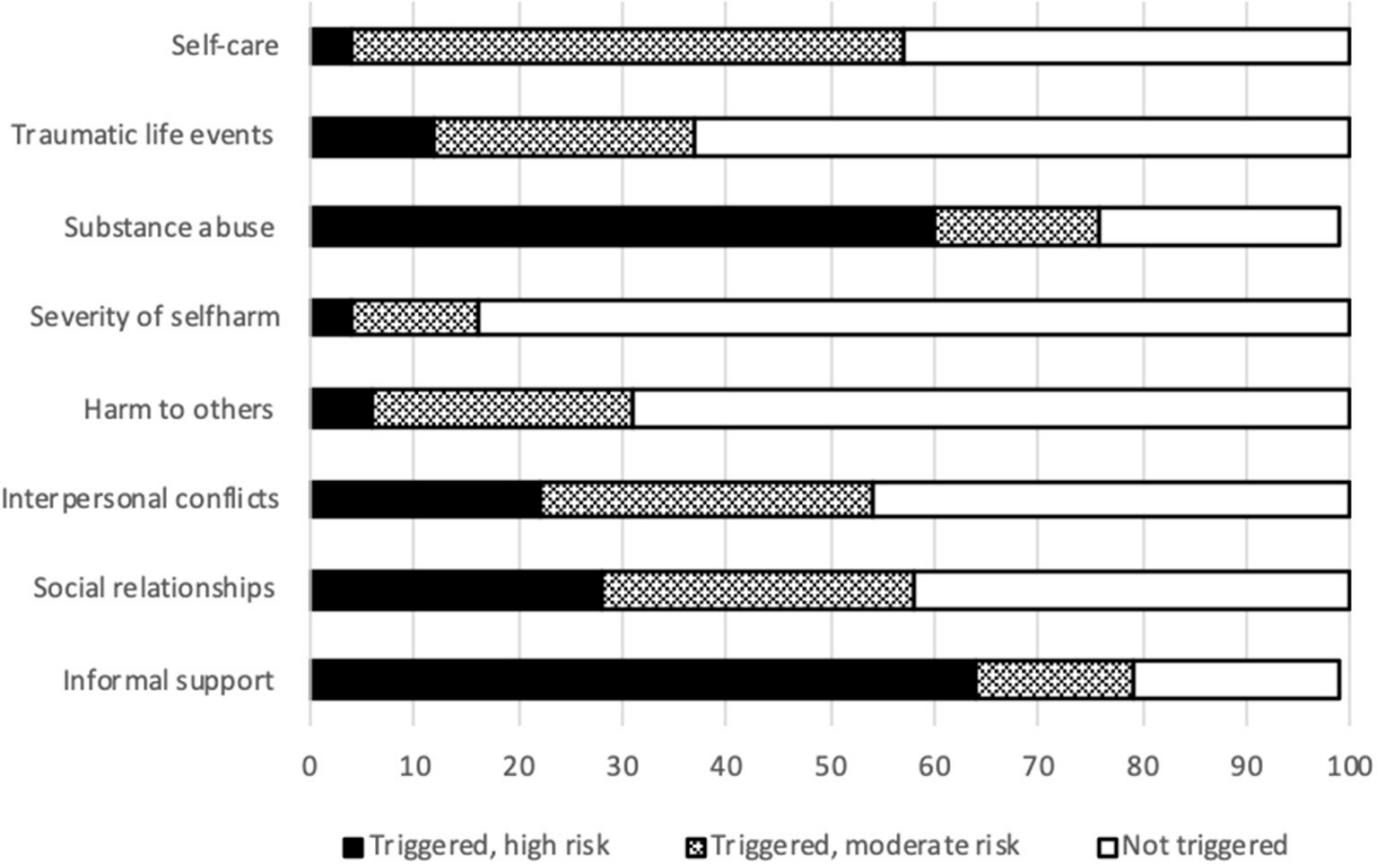
Triggering rates for eight safety CAPs.

The interRAI CAPs are designed to translate observations into meaningful risk indicators. Figure 5 shows the results on some CAPs reporting on behavior and performance. Limited personal autonomy is common (57.0%). By contrast, insufficient selfcare only sometimes produces severe risk (4.0%).

## Discussion

The HOP-TR study is a recent large study among Dutch HS users. This paper presents the study sample and provides insight into HS users' physical and mental health.

Most HS users were males, low educated, with a migration background. Additionally, even more important in the light of recovery, most HS users were long-term or intermittently homeless. The assessment of mental and physical discomfort shows extensive multi-domain disabilities. Mental symptoms in daily life are most prominent, but physical health problems are also prevalent. Health problems tend to co-occur. Almost all subjects have problems in two domains (94.7%); a large subgroup has comorbidity in three or four domains. Mental illness and addiction frequently co-occur (78.0%). Comorbid severe chronic physical conditions are present in more than half the HS users (59.2%); intellectual impairments in 39.9%. These results illustrate the presence of health problems in different interdependent domains in Dutch HS users. Symptoms and disabilities trigger each other; for example, past traumas impact on daily life. Although most subjects are capable of making independent, safe, and reasonable decisions, the accumulation of health problems and vicissitudes limits their ability to find adequate care in modern over organized and extensive siloed care systems.

The sample profile confirms prior findings on shelter use in high-income countries. In the early nineties, Kuhn and Culhane were the first ones to cluster service users on administrative data (33). In the shelters of New York City and Philadelphia, they identified three clusters: transitional, episodic and chronic shelter users. The prevalences of SMI, substance use and medical problems were substantially higher in subjects of the episodic and chronic clusters. The chronic cluster (10%) consumed half of all shelter days. Later, replications of this approach in Canada and Denmark revealed similar patterns (34, 35). The high share of long-term or intermittently homeless subjects in this sample supports that in high-income countries, with elaborated care systems, homelessness mainly affects people with complex needs (35).

The epidemiological homelessness literature reports an accumulation of risk factors and exclusion mechanisms (2, 35–37). The relevance of (severe) mental illness to the risk of marginalization is generally accepted (5, 38–42). In the early nineties, homeless people with SMI and addiction were part of the “special populations” targeted in case management care such as Assertive Community Treatment (ACT) or Intermediate Case Management (ICM) (43, 44). Housing First, including ACT or ICM, proved to be successful for mentally ill homeless individuals (45). Sylvestre, Nelson and Aubry consider housing as a starting point for social integration, community participation, recovery and citizenship (46).

Still, specialized care is organized in siloed services, which are insufficiently capable to serve complex patterns of problems and needs in marginalized groups (47–49). Denmark has one of the largest Housing First (HF) programs in Europe, but this still only serves 5% of homeless (50). Even among chronic homeless, HF with some form of case management, is marginally offered (11%). In the Netherlands, Slockers et al. used a register based 10-year follow up study among HS users in Rotterdam to evaluate the municipal policy before and after the start of the national Dutch homelessness strategy (51). Despite improvement of the living conditions, mortality rates did not decrease. Regarding the need of additional measures in addition to housing and local services, they recognized the intermittent or persistent character of the homelessness.

Recently, Rosen, Gill, and Salvador-Carulla addressed the failure of mental healthcare systems to meet complex multisectoral care needs, such as in homeless populations (52). They revised the literature on critical elements at the individual and regional/national level. They argued to reframe community healthcare in a healthcare ecosystem approach, targeted at balanced care (53) and rooted on the keystones of community mental health (person-centeredness, recovery, human rights, challenging stigma and discrimination).

How to create better conditions – in addition to housing – for social integration, community participation, recovery and citizenship? In the Netherlands, the HOP-TR study started a public health dialogue at different ecosystem levels. The regional assessments fulfilled the information needs of local service administrators. In each region, the transparent results stimulated reflection and discussion about care provision, network cooperation, care access, and quality of care. At the same time, the regional dialogues pushed the national debate and kept the meta-snowball rolling. More local authorities commissioned a study of their own local situation. In some cities the data were used to forge long-term commitment for care improvement.

This paper presents the extent of health-related needs of the Dutch HS users beyond housing. The results underline the need for an integrated, rights-based, 3-D recovery-oriented public health approach. The significant part of previously homeless subjects emphasizes the urgency of comprehensive care. The high prevalence of SMI/EPA underscores that integrated mental health is a cornerstone service for HS users in need of a place to live in the community.

The assessment strategy in the HOP-TR study was designed to explore new perspectives on recovery in marginalized populations with interdependent needs. The strategy adds new elements to the (predominantly descriptive) homelessness literature.

First, mental health is assessed transdiagnostically, using the HS users' perspective as the primary data source. The strategy shows a high prevalence of anxiety, trauma and depression. Still, the trend in most prevalence estimates is comparable to the literature on chronic and intermittent homeless populations (5, 12, 23, 38, 39). Additionally, it introduces the SMI/EPA concept as a quality indicator of the need of integrated care. Finally, it comprehensively assesses symptomatic, social, and existential health aspects relevant to recovery in homeless populations. It comprises both the perspective of HS users and professionals, and helps us to understand how HS users engage with services.

Limitations – In this study, a dual snowball sampling was applied to recruit a saturated sample of settings and subjects. In comparison to reference populations, the subjects in this study were older, lower educated and less often migrants. The higher age might point to a real trend in the population, but might also be an artifact of differences in settings and recruitment procedures. The lower prevalences of physical symptoms (Table 2), compared to mental symptoms (Figure 1), suggests underreported physical symptoms. The cross-sectional design overrepresented individuals with complex health problems by assessing the most needing individuals who stay in the facilities the longest time. Considering the hidden nature of this population, the HOP-TR recruitment strategy is the best possible approach to comprehensively assess a representative sample of the Dutch adult HS users in 2015–2017.

Also, all data were collected in single interviews by a single researcher. Corrobative medical information was not available. Four out of five subjects had not visited any physician within the last 3 months. This shortcoming was cared for using individual assessments by a researcher with an MD professional background. Single person assessments might induce a bias but offer best health estimates of a hidden part of the Dutch HS population. Finally, the data quality is limited to information collected during a single encounter. Better care access and additional checkups certainly would provide more reliable descriptive health data.

## Conclusions

This paper presents factual empirical health data on a representative sample of Dutch HS users. Most subjects were long-term and intermittently homeless. The prevalences of physical and mental health problems, addiction, and intellectual impairments are comparable to other publications in homeless populations. The key results are the high rates of health problems, their interrelations, and reciprocity. Almost all HS users had health problems in two or more health domains (94.7%). A significant part had severe mental health problems (72.5%).

The high comorbidity in this difficult to access populations emphasizes the need for integrated, rights-based and 3-D recovery oriented public health services. The HOP-TR approach appears useful to foster the dialogue at different healthcare ecosystem levels how to improve care.

## Data Availability Statement

The raw data supporting the conclusions of this article will be made available by the authors, without undue reservation.

## Author Contributions

CE, PP, KV, and PD participated in the conceptual design of the study. CE drafted the manuscript, collected the data, and performed the analyses. All the authors critically revised the manuscript, contributed to interpretation of the data, read, and approved the final version of the manuscript.

## Conflict of Interest

The authors declare that the research was conducted in the absence of any commercial or financial relationships that could be construed as a potential conflict of interest.

## Footnotes

^1^“Cohortonderzoek Dak- en thuislozen in de G4” (Cohortstudy of homeless people in the G4); the G4 acronym indicates the four big Dutch cities: Amsterdam, Rotterdam, The Hague and Utrecht.

^2^The Dutch acronym to indicate SMI.
