# Supplementary material for: Health Patterns Reveal Interdependent Needs of Dutch Homeless Service Users
Source: Front Psychiatry. 2021 Mar 25;12:614526. doi: 10.3389/fpsyt.2021.614526 (PMC8027245; doi:10.3389/fpsyt.2021.614526)
Supplement: Supplementary file 1 [file Table_1.DOCX]

**Supplementary table 1. Comparison of HOP-TR sample with CODA-G4 sample**

|  | **HOP-TR** | | **CODA-G4** | |  |
| --- | --- | --- | --- | --- | --- |
| **Total sample** | n = 436 | | n = 513 | |  |
| Mean age (years) | 45.0 |  | 40.3 |  | t = 7.69, p < 0.01 |
| Standard deviation | 13.09 |  | 13.29 |  |  |
| Age range | 18-75 |  | 18-71 |  |  |
| Male | 353 | 81.0% | 391 | 76.2% | χ^2^ (1) = 3.13, p 0.08 |
| Female | 83 | 19.0% | 122 | 23.8% |  |
| Native | 209 | 47.9% | 202 | 39.4% | χ^2^ (1) = 7.03, p 0.01 |
| Migration background | 227 | 52.1% | 311 | 60.6% |  |
| **Adults** | n = 398 | | n = 410 | |  |
| Mean age (years) | 45.0 |  | 40.3 |  | t = 5.74, p < 0.01 |
| Standard deviation | 11.55 |  | 11.72 |  |  |
| Age range | 23-75 |  | 23-71 |  |  |
| Male | 329 | 82.7% | 329 | 80.2% | χ^2^ (1) = 0.81, p 0.37 |
| Female | 69 | 17.3% | 81 | 19.8% |  |
| Native | 192 | 48.2% | 164 | 40.0% | χ^2^ (1) = 5.57, p 0.02 |
| Migration background | 206 | 51.8% | 246 | 60.0% |  |
| **Young adults** | n = 38 | | n = 103 | |  |
| Mean age (years) | 20.1 |  | 20.2 |  | t = 0.41, p 0.68 |
| Standard deviation | 1.29 |  | 1.31 |  |  |
| Age range | 18-22 |  | 18-22 |  |  |
| Male | 24 | 63.2% | 62 | 60.2% | χ^2^ (1) = 0.10, p 0.75 |
| Female | 14 | 36.8% | 41 | 39.8% |  |
| Native | 17 | 44.7% | 38 | 36.9% | χ^2^ (1) = 0.72, p 0.40 |
| Migration background | 21 | 55.3% | 65 | 63.1% |  |

Supplementary table 1 compares background characteristics of the HOP-TR sample with the CODA-G4 sample (<http://www.codag4.nl/Coda_G4_rapporten,78>; table 2.1, p. 17). The CODA-G4 study is a multisite cohort study for monitoring the Dutch national action plan to end homelessness, which ran from 2011-2013. Subjects were recruited in municipal offices and in homeless services.

The CODA-G4 report describes the background characteristics of young adults (18-22 years) and adults (23 years and older) separately. The numbers of the subsamples were used to calculate mean age, gender, and migration background of the total CODA-G4 sample, presented in the upper section of supplementary table 1. The standard deviations of the age distributions in the CODA-G4 sample were unknown but calculated under the assumption of equal distributions.

Comparison of the HOP-TR sample with the CODA-G4 sample reveals that subjects in the HOP-TR sample are older. Also, the share of subjects with a migration background is significantly lower. Observed differences in gender can be explained by chance.

**Supplementary table 2. Comparison of the HOP-TR sample with CBS samples**

|  | **HOP-TR**  2015-2017 | | **CBS**  2015-2017 | |  |
| --- | --- | --- | --- | --- | --- |
| Male | 353 | 81.0% | 26333 | 82.3% | χ^2^ (1) = 0.52, p.47 |
| Female | 83 | 19.0% | 5667 | 17.7% |  |
| Age 18 - 29 | 84 | 19.3% | 10500 | 33.0% | χ^2^ (6) =15262 p <0.01 |
| Age 30 - 49 | 203 | 46.6% | 15867 | 49.3% |  |
| Age 50 - 65 | 129 | 29.6% | 5600 | 17.3% |  |
| Age 65 and older | 20 | 4.6% | 0 |  |  |
| Native | 209 | 47.9% | 14300 | 44.7% | χ^2^ (1) = 14105, p <0.01 |
| Migration background | 227 | 52.1% | 17700 | 55.3% |  |
| Education low | 359 | 82.3% | 20143 | 65.5% | χ^2^ (4) = 54.7, p <0.01 |
| Education middle or high | 65 | 14.9% | 9378 | 30.5% |  |
| Education high | 12 | 2.8% | 1230 | 4.0% |  |

Supplementary table 2 compares background characteristics of the HOP-TR sample with the yearly samples underlying the Dutch homelessness estimates of Netherlands Statistics (CBS). In 2015-2017, the number of homeless people in the Netherlands fluctuated from 31000 (2015), and 30500 (2016), to 34500 (2017). The results in the right column reflect the means of the corresponding numbers in 2015-2017.

Supplementary table 2 shows that gender in both samples is the same. In comparison to the CBS samples, subjects in the HOP-TR sample are older, more often native, and low-educated. All differences are highly significant.
